# A Bibliometric and Visual Analysis of Cancer Screening Based on the Web of Science Core Collection Database

**DOI:** 10.34172/ijhpm.8554

**Published:** 2025-09-15

**Authors:** Wanrong Dai, Baixu Wu, Jie Sun, Wuping Shuai, Shuying Han

**Affiliations:** ^1^Department of Clinical Pharmacy, the First Affiliated Hospital, Zhejiang University School of Medicine, Hangzhou, China.; ^2^CUHK Business School, Hong Kong, China.

**Keywords:** Cancer Screening, Bibliometrics, Visualization Analysis, Artificial Intelligence (AI)

## Abstract

**Background:**

This study aimed to systematically analyze the current research status, development trends, collaborative networks, and hot topics in the global cancer screening field using a bibliometric method. It sought to reveal the contributions and influences of different countries and institutions and explore potential directions for future research, providing a comprehensive basis for academia and policy-makers to optimize cancer screening strategies.

**Methods:**

We searched the Web of Science Core Collection on October 15, 2023, using TS = (cancer screening) and DT = (Article), with no restrictions on the language or publication year. Only original research articles directly related to cancer screening were included; abstracts, comments, and non-research literature were excluded. VOSviewer was used for co-occurrence analysis to assess research status and hotspots. CiteSpace analyzed annual publication trends, collaboration networks among countries, institutions, journals, authors, and keywords.

**Results:**

A total of 5223 articles were retrieved, showing a continuous growth trend in annual publication volume. The USA had the highest output (2418), followed by the UK and the Netherlands. Harvard University was the most productive institution (183). *Cancer* published the most articles (120), while the *New England Journal of Medicine* had the most citations (7991). High-frequency keywords included screening (987), colorectal cancer (CRC) (783), mortality (680), women (671), and breast cancer (BC) (669). Cluster analysis revealed seven main research themes: CRC, cervical cancer (CC), lung cancer (LC), BC, cancer screening, human papillomavirus (HPV) vaccination, and lynch syndrome. Hot topics included LC screening and adherence. Future research may increasingly focus on artificial intelligence (AI) and deep learning (DL), aiming to introduce new technologies and optimize screening strategies to improve efficiency and early diagnosis.

**Conclusion:**

Research on cancer screening is rapidly advancing, with the USA leading in productivity and influence. Current research mainly focuses on CRC, CC, LC, and BC.

## Introduction

 Cancer is a leading global culprit of death. A common characteristic of many cancers is that favorable outcomes are often achieved when the disease is detected at a local stage, accompanied by early treatment in the natural history of the disease. The purpose of cancer screening is to reduce the incidence of late-stage diseases by detecting malignant tumors or their precursors (eg, polyps before colorectal cancer [CRC], intraepithelial neoplasia before cervical cancer [CC]) during the early stages before symptoms appear, allowing patients to initiate treatment earlier.^1^ The methods of cancer screening have evolved with the iterative development of detection techniques. In the field of fecal testing for CRC, for instance, Van Deen^2^ in 1864 first utilized the guaiac-based Hemoccult (SmithKline Diagnostics, Sunnyvale, California) test to investigate hemoglobin in human feces. In 1901, Boas^3^ recommended the guaiac test as a diagnostic test for gastrointestinal cancer. In the mid-1960s, Greegor^4^ applied the guaiac fecal occult blood test (FOBT) in screening CRC. Recently, quantitative immunochemical FOBT has gained wide attention, being used for CRC screening in some Western countries. FOBT has progressed from chemical testing to immunochemical testing, further developing from qualitative detection to quantitative detection.

 Currently, screening for certain cancers, such as CRC, prostate cancer, breast cancer (BC), and CC, can substantially reduce disease-specific mortality and overall cancer mortality.^5^ However, the effectiveness of screening for early-stage cancer or cancer precursors is not always as evident for other types of cancer. For example, in the UK Collaborative Trial of Ovarian Cancer Screening, a single transvaginal ultrasound examination of asymptomatic women did not reduce mortality caused by ovarian cancer, which is not suitable as a standalone screening method for ovarian cancer as a result.^6^ Additionally, screening tests are preventive intervention measures performed on asymptomatic and healthy populations, which may lead to overdiagnosis and unnecessary intervention when the disease incidence is low.^7^ When the value of screening is convincing and the benefits outweigh the harms, authoritative organizations issue guidelines and recommendations, developing policies that support cancer screening.^1^

 Given the crucial role of cancer screening in public health and the rapid growth of related research in recent years, we have noticed that there is currently a lack of bibliometric research that can systematically outline the development pattern of cancer screening from a holistic perspective. Most existing research focuses on a single type of cancer, a single screening method, or specific regions, making it difficult to fully reveal the knowledge evolution path and collaborative network in this field, which limits the scientific integration of scientific research resources and the formulation of strategies. In addition, current research is mostly focused on specific practical applications, and there is a lack of systematic analysis to reveal the structural gaps in the current knowledge system in this field from a macro level. There is also a lack of quantitative refinement of research hotspots, development trends, and future directions. This limitation is particularly evident in the context of emerging technologies, and there is an urgent need for trend analysis based on global literature to provide strategic references for researchers and policy-makers. Bibliometrics, as an effective scientific measurement tool, can quantitatively and visually analyze published literature to demonstrate the current research status and future directions in a certain field.^8^ This study utilized data from the Web of Science Core Collection database, using bibliometrics and visualization methods to systematically analyze the evolution process, hot topics, core authors, research institutions, and international cooperation patterns of cancer screening research from a macro level. Our research not only provides an overview of the development and future directions of cancer screening for the academic community, but also provides data support and theoretical basis for optimizing screening strategies, allocating research resources, and formulating health policies at the practical level, thereby enhancing more scientific and efficient cancer screening work on a global scale.

## Materials and Methods

###  Data Source and Search Strategy

 The Web of Science Core Collection was the data source, with a search date of October 15, 2023, and the keywords as TS = (cancer screening) and DT = (Article). There were no restrictions on the language and publication year of the article, and the article type is limited to “Article.” We exported the full record of search results and cited references as a plaintext format file (including complete fields such as title, author, abstract, institution, keywords, and references) for subsequent processing and analysis using visual analysis software.

###  Data Cleaning and Filtering 

 The retrieved literature records underwent preliminary cleaning and screening to ensure accurate and effective analysis of the data. The main steps are as follows:

 De-duplication processing: The literature management software or Excel was utilized to de-duplicate exported data and remove duplicate entries during the retrieval process.

 Exclusion of irrelevant literature: Two researchers independently read the title and abstract, and excluded articles that contained keywords such as “cancer” or “screening” but were unrelated to the topic of cancer screening.

 Exclusion of non-original research literature: We excluded conference abstracts, review articles, book reviews, editorials, and other non-original research literature to ensure that the remaining literature was original research articles.

 After cleaning and screening, a literature dataset for visual analysis was obtained, with a total of 5223 records. All screening steps were independently completed and cross-validated by the researchers to ensure consistent screening criteria and avoid irrelevant literature affecting the analysis results.

###  Data Analysis and Visualization

 To comprehensively elucidate the current development status and research hotspots in the field of cancer screening, this study used various bibliometric analysis software to analyze and visualize the cleaned dataset. The main software and its applications are as follows:

 VOSviewer (version 1.6.19): VOSviewer was used for building co-occurrence networks and clustering analysis, specifically applied in collaborator networks between countries or institutions, keyword co-occurrence networks, and co-citation networks for journals/authors. When conducting keyword co-occurrence analysis, a threshold was set for the frequency of keyword occurrence (for example, only retaining keywords that appear ≥ 5 times) to exclude rare entries. We chose LinLog or VOS layout for network layout, used VOSviewer’s built-in modular clustering algorithm to automatically partition topic clusters, and presented literature volume or link strength through node size and colors in the visualization graph.

 CiteSpace (v6.2.4): The CiteSpace was employed for analyzing reference co-citation networks, keyword time zone maps, and burst analysis. The clustering algorithm adopted the default Louvain community discovery algorithm, and the clustering labels were usually generated based on the log likelihood ratio method. In the study, the quality of clustering results was also evaluated, such as the modularity Q value (Q > 0.3: Clustering significance) and the average silhouette coefficient S (S > 0.5: Clustering reliability). The knowledge graph generated through CiteSpace visually displayed the evolution trajectory of the field and the transfer of research hotspots.

 Microsoft Excel: Microsoft Excel was utilized for data organization and basic statistical analysis, including drawing annual publication trend charts, compiling literature output of major countries/institutions, author and journal distribution, and citation frequency distribution. By processing raw exported data through Excel, descriptive statistical results can be quickly obtained, providing data support for visual display.

 SCImago Graphica (v1.0.36): SCImago Graphica was used for creating interactive visual charts. For example, SCImago Graphica was used to draw string plots or geographical distribution maps of national/regional cooperation networks to visually display the strength of cooperation and network structure between countries. This software generated advanced visualizations such as chord diagrams through drag and many kinds of charts, helping to reveal collaborative relationships between multiple countries.

## Results

###  Publication Trend

**Figure 1:**
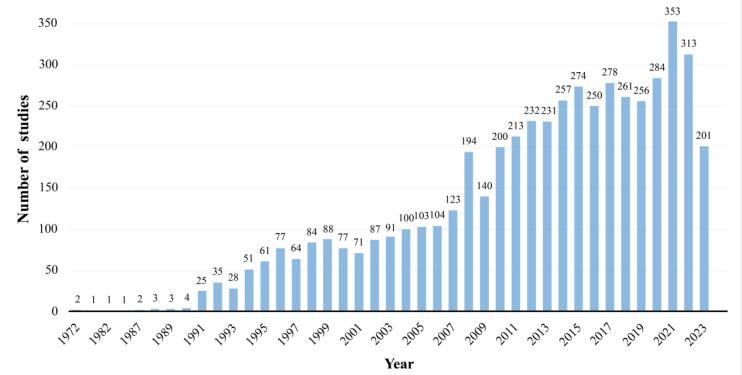
Annual Number of Publications.

 We retrieved 5223 articles. As shown in Figure 1, the first study was published in 1972. The number of publications had steadily increased since then. Before 1991, there were relatively few studies of cancer screening, with fewer than 5 articles published each year. The volume of publication gradually increased after 1991. Additionally, starting from 2004, the annual publication count exceeded 100 articles. From 2008 onwards, there has been considerable growth in annual publication numbers, reaching a peak of 353 articles in 2021. In 2022, there was a slight decline in the quantity of publications compared to 2021, with a total of 313 related studies published.

###  Global Distribution and Collaboration

 In terms of publication volume, Table displays the top 10 countries, including 6 European countries, 2 North American countries, Australia, and China. The USA, the UK, and the Netherlands had the highest number of published articles and the highest total link intensity, indicating their important influence in the field of cancer screening.

**Table:** Top 10 Countries Contributed to Publications

**Rank**	**Country**	**Documents**	**Citations**	**Total Link Strength**
1	USA	2418	97 817	1184
2	UK	593	22 249	746
3	Netherlands	410	18 474	541
4	Canada	355	18 178	362
5	Australia	336	11 301	386
6	China	321	7386	303
7	Italy	258	7711	421
8	France	246	8118	439
9	Germany	228	7325	441
10	Spain	147	4556	283

**Figure 2:**
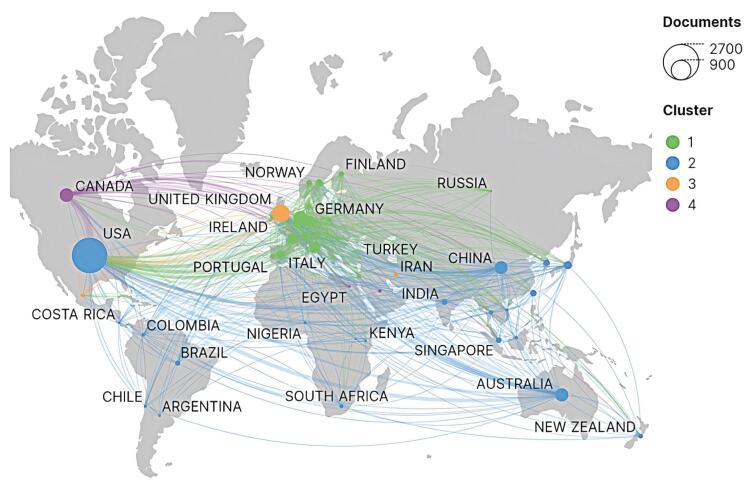
The World Distribution and Collaboration of Cancer Screening.

Figure 2 displays a map of country/region collaborations in the field of cancer screening. The size of the nodes reflects the quantity of publications, while the lines between nodes reflect collaborations. Through co-authorship analysis, we divided countries/regions into different clusters according to VOSviewer, with nodes of different colors representing different clusters. The USA collaborated closely with other countries. European countries had close collaboration with Asian and North American countries. Canada frequently collaborated with the USA and European countries (Figure 2).

###  Research Institutions

 Harvard University (183 articles) was the most prolific institution in issuing research, followed by the University of Washington (165 articles) and the University of California, San Francisco (117 articles). In terms of publication volume, 7 out of 10 institutions were from the USA. The other 3 institutions were the University of Toronto in Canada, the University of Sydney in Australia, and Erasmus University Medical Center in the Netherlands.

**Figure 3:**
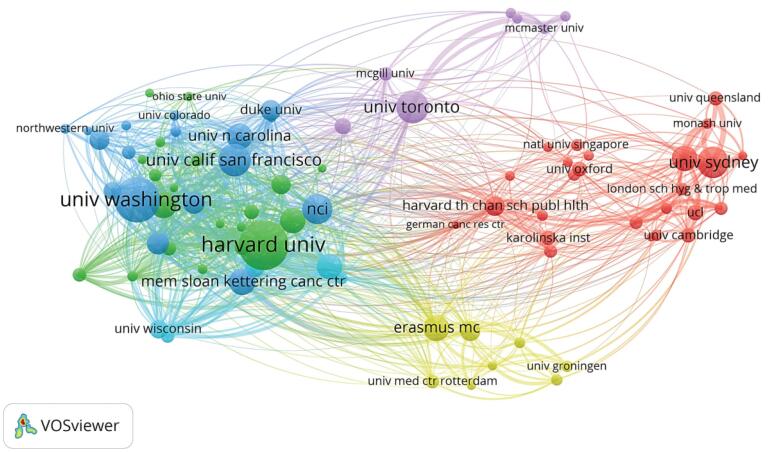
Network Among Institutions.

 Based on the VOSviewer, a visualization was created for 83 institutions (minimum publication count ≥30). A collaboration network was constructed based on the publication quantity and relationships of each institution. Nodes of different colors in Figure 3 represent different clusters. There was close collaboration within countries such as the Netherlands, Canada, and the USA, as well as among universities and institutions within each country. Institutions such as the University of Sydney, the University of Queensland, and Monash University in Australia actively collaborated with institutions in the UK, such as the London School of Hygiene & Tropical Medicine, the University of Oxford, and the University of Cambridge, as well as institutions like the National University of Singapore in Singapore and Karolinska Institutet in Sweden.

###  Journals and Co-cited Journals

*Cancer* was the journal with the highest number of documents in the field of cancer screening (120 documents), followed by *PLoS One* (109 documents), *International Journal of Cancer* (84 documents), *Cancer Epidemiology Biomarkers *&* Prevention* (63 documents), and *Annals of Internal Medicine* (60 documents). Among the top 10 journals, *Annals of Internal Medicine* had the highest impact factor (IF = 39.2), followed by the *British Journal of Cancer* (IF = 8.8).

 Three out of 10 co-cited journals had been cited more than 5000 times. The most-cited journal was the *New England Journal of Medicine* (frequency = 7991), followed by *JAMA-Journal of The American Medical Association* (frequency = 5683) and *Annals of Internal Medicine* (frequency = 5288). Additionally, the impact factorof* Lancet* was the highest (IF = 168.9), followed by the *New England Journal of Medicine* (IF = 158.5).

###  Research Hotspots and Frontal Analysis

 As a summary of the research content, keywords can reflect the key information of the articles. By co-occurrence analysis of keywords, we immediately identified the research hotspots in a specific field. In addition to “screening” (987), and “cancer” (652), the frequently occurring keywords included “CRC” (783), “mortality” (680), “women” (671), “BC” (669), “risk” (666), “CC” (564), and “colonoscopy” (408).

 A cluster analysis of keywords was conducted using CiteSpace, resulting in 7 clusters: #0 CRC, #1 CC, #2 lung cancer (LC), #3 BC, #4 cancer screening, #5 human papillomavirus (HPV) vaccination, and #6 lynch syndrome. The smaller cluster number indicated the more keywords it contained. Each cluster was composed of closely related terms. The Q-value was 0.3594 (Q-value: The modularity value of the clustering. Q>0.3: Significant clustering results). The S-value was 0.739 (S-value: The average silhouette value of the clustering. S>0.5: reasonable clustering results). The Q- and S-values indicated effective classification and clustering.

**Figure 4:**
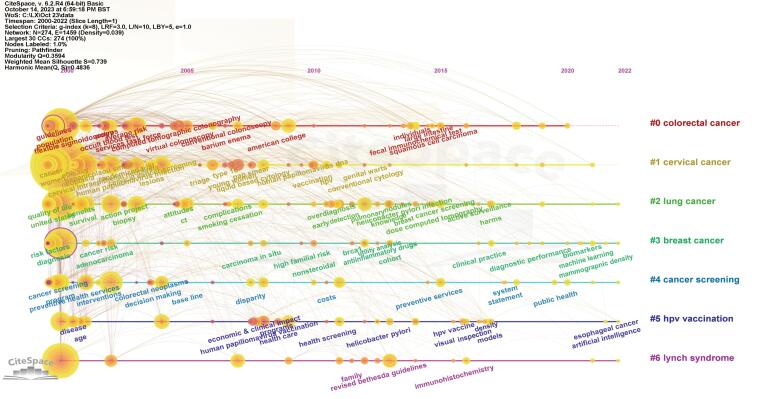
The Clustering Timeline of Keywords.

Figure 4 shows the timeline of keyword clustering, showing the evolution of research topics in the field of cancer screening. The horizontal axis represents time, the vertical axis represents various cluster topics, and each horizontal line represents the evolution path of keywords within a cluster. Keywords before 2005 were mainly concerning guidelines, population, cancer, quality of life, health service, etc, focusing on the basic concepts of cancer screening and health services. From 2005 to 2015, the research focus shifted towards keywords such as chromocopy, BRCA1, cohort, dispersion, economic & clinical impact, reflecting the emphasis on specific screening techniques and population research. The keywords that emerged after 2015 included machine learning, artificial intelligence (AI), immunohistochemistry, etc, indicating that the field is introducing new technologies and methods, which is an important direction for future research.

**Figure 5:**
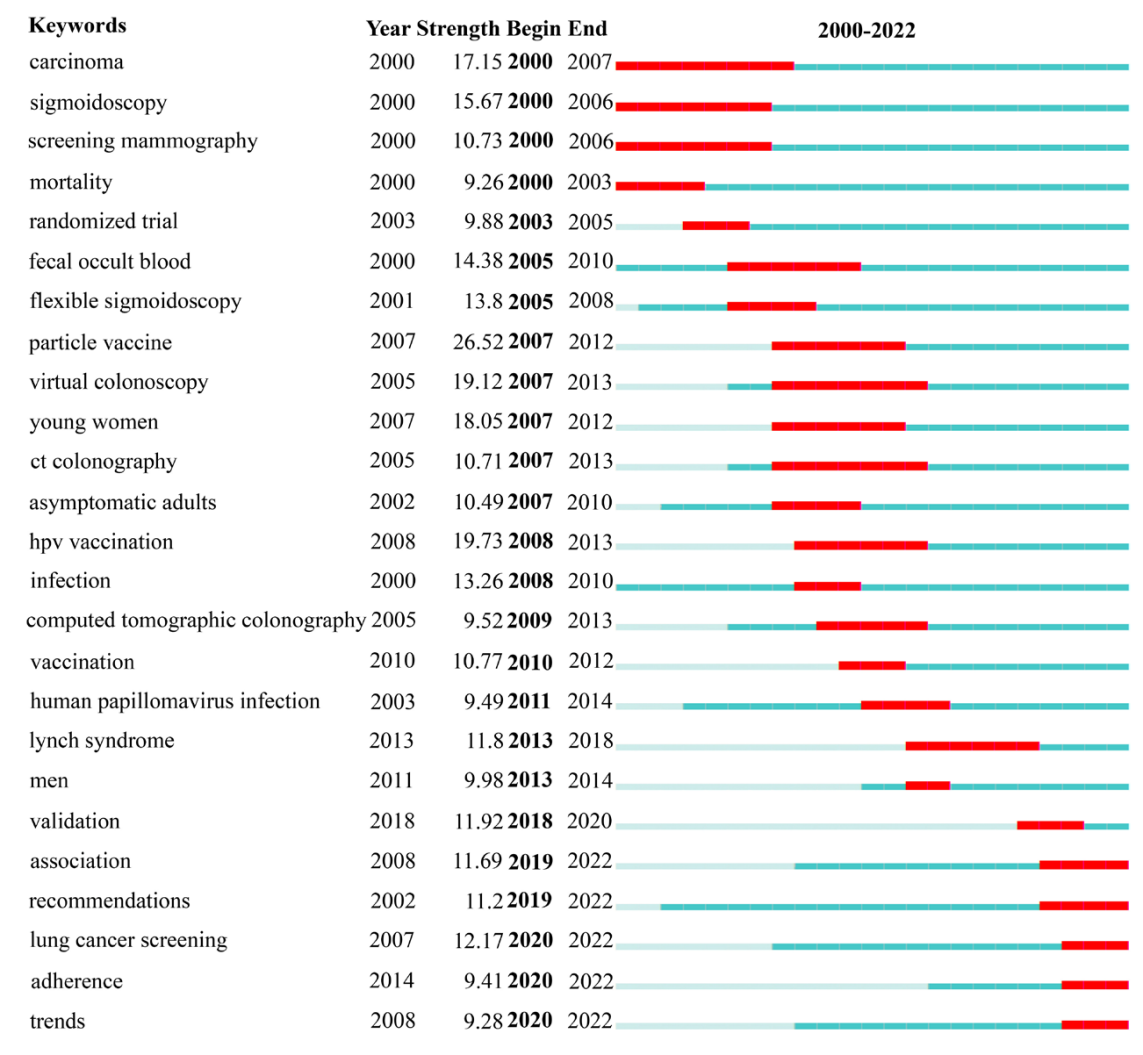
Top 25 Keywords With the Strongest Citation Bursts.

 In addition, keyword burst analysis reflected the trend of certain terms being frequently cited during a specific period and was commonly used to identify research frontiers. Figure 5 shows the top 25 keywords with the strongest outbreaks from 2000 to 2022. The blue line represents the timeline, and the red portion on the blue timeline represents the outbreak period (start year, end year, and duration of the outbreak). The citation explosion of the Particle vaccine (26.52) was the strongest, followed by HPV vaccination (19.73) and virtual chromocopy (19.12). The hot topics in the early 2000s included screening mammography and mortality. In the mid-term, the focus was on computed tomography and HPV infection. In recent years, keywords such as LC screening, adherence, and trends emerged, indicating current research hotspots and great potential for development in the future.

## Discussion

 We herein employed bibliometric methods and visualization tools to analyze over 5000 studies of cancer screening. Based on publication volume, it was observed that prior to 1991, there were relatively few publications in this field, indicating an early stage of the research. However, from 2008 onwards, the field experienced rapid development. Through statistical analysis of the number of publications in different countries/regions and institutions, we found that the USA contributed remarkably more publications and collaborations with other countries/regions compared to other nations. Additionally, in terms of publication volume, 7 out of 10 institutions were based in the USA, with the 3 most productive institutions being the USA universities, including the University of Washington, Harvard University, and the University of California, San Francisco. These results highlighted the substantial contributions made by the USA in the field of cancer screening, establishing its leading position. Furthermore, several developed countries in Europe also made important contributions to this field. There was close collaboration and academic exchange among countries and institutions, aiding in overcoming academic barriers and ultimately fostering further advancements in this field.

 Journals serve as important vehicles for presenting academic information and disseminating knowledge. Among all the journals publishing relevant articles, *Cancer* contributed the largest number of publications, suggesting its status as the most popular journal in this research field, followed by *PLoS One*. In terms of cited journals, the *New England Journal of Medicine* ranked first with the highest citation frequency, signifying its crucial academic influence in the field. Other frequently cited journals, such as* Annals of Internal Medicine*, *JAMA-Journal of The American Medical Association*, and *Lancet* were high-quality international journals, proffering support to the field of cancer screening.

 Keywords are the essence and centerpiece of a paper, encapsulating and representing the main content, academic ideas, and ultimate conclusions. By analyzing a large number of keywords in papers, researchers can quickly grasp the hot topics and evolution in a research field. Visualizing the keywords in articles related to cancer screening, popular keywords, in addition to “screening” and “cancer,” included “CRC,” “mortality,” “BC,” “CC,” and “colonoscopy,” indicating that BC, CC, and CRC were most frequently studied in this field. Colonoscopy was a frequently studied screening method, and mortality was a commonly used research indicator.

 Cluster analysis based on keywords identified 7 clusters, including CRC, CC, and LC.

 As an invasive malignant tumor developed from the colonic and rectal mucosa, the majority of CRC is classified as adenocarcinoma.^9^ Multiple subtypes have been proposed for the initiation of polyps and cancer progression, including the adenoma-carcinoma sequence, where adenoma serves as the precursor to cancer, and the serrated polyp-carcinoma sequence, where the precursor to cancer is the sessile serrated lesion (SSL, or serrated polyp-carcinoma sequence).^9^ Most adenomas and SSL polyps can be detected through endoscopy, thus being suitable for polypectomy and cancer prevention.^10^ Adenomas, SSL, and CRC, with a tendency to bleed, can shed abnormal cells with detectable molecular markers, making them detectable through fecal-based testing.^10^ Emerging blood-based tests can detect genetic and epigenetic changes related to polyps and cancer that leak into the circulation.^11^ These characteristics make CRC an ideal screening target.

 The participation rate of colonoscopy is reduced than non-invasive tests such as fecal immunochemical testing (FIT), though it is the standard for CRC screening and diagnosis.^12^ Fecal-based testing includes blood detection in the stool using FIT and the detection of DNA mutations and methylation using the multi-target stool DNA test, also known as Cologuard.^10^ A well-applied blood test, Epi proColon, can detect methylated SEPTIN9 DNA.^10^ Radiologic examinations such as colon capsule endoscopy and computed tomographic colonography aim to visualize and identify colon polyps and cancer.^10^ Additionally, screening tests targeting novel biomarkers, such as fecal bacterial markers and microRNAs, are also under development.^13-15^ Previous studies have indicated that alternative tests, compared to colonoscopy and FIT screening, are not cost-effective.^16-18^ A microsimulation screening analysis conducted by Peterse et al^19^ in the MISCAN-colon model demonstrated that Epi proColon is more cost-effective compared to colon capsule endoscopy, computed tomographic colonography, and Cologuard. For individuals unwilling to take FIT or colonoscopy, annual screening with Epi proColon is the preferred test.^19^

 Papanicolaou smear, also known as cervical cytological test, is the most frequently utilized test for early detection of CC.^20^ The Papanicolaou smear collects and examines exfoliated cervical cells to analyze abnormal cells, which may indicate cervical precancerous lesions and early-stage CC.^21^ HPV infection is proven to be a prerequisite for CC, which is a scientific breakthrough that greatly changed CC screening.^22^ Precancerous lesions and invasive cancer can be induced by a lasting infection with high-risk HPV strains.^23^ Therefore, vaccines and new screening methods have been promoted based on the discovery of the association between HPV and cancer. Current guidelines recommend using cytology, HPV testing, and co-testing for CC screening.^24^ In a recent systematic review on the economic evaluations of CC prevention strategies in low- and middle-income countries, HPV testing is proven to be cost-effective compared to smear tests for women in these areas.^25^

 The HPV vaccine is indicated for the prevention of specific subtypes of HPV infections, aiming to prevent diseases such as CC induced by persistent infection with high-risk HPV strains.^26^ All vaccines protect against the highest-risk subtypes of CC, namely HPV-16 and HPV-18 infections. Additionally, the HPV vaccine also prevents the tumorigenesis of CC, anal cancer, vaginal cancer, and some oropharyngeal cancers.^27,28^ It is noteworthy that the vaccination rates for HPV in low- and lower-middle-income countries are considerably lower compared to high- and upper-middle-income countries.^29^ The research indicated that conducting HPV screening every 5 years and administering vaccination are the most economically effective measures for CC prevention in China.^30^

 A high proportion of LC patients present with advanced-stage disease at diagnosis. Despite advancements in treatment, the mortality rate remains high. Chest X-rays and sputum cytology tests have been applied as early screening tests. Although these methods have shown improved survival rates in detecting LC patients, they have not exhibited a decrease in LC-specific mortality.^31,32^ Large randomized controlled trials, such as the National Lung Screening Trial (NLST)^33^ and the subsequent NELSON (Nederlands-Leuvens Longkanker Screenings Onderzoek) trial^34^ conducted in 2011, have established that LC screening by low-dose CT can decrease cancer-specific mortality.

 Mammography, which utilizes images of the breast with low-dose two-dimensional X-ray, is the most prevalently employed test for assessing BC in the early stage, aiming to detect suspicious growths that cannot be felt.^35^ Multiple evaluations of existing data from randomized controlled trials over the past 30 years have demonstrated that routine mammography can reduce the relative risk of BC-related deaths.^36^ Breast magnetic resonance imaging is another commonly employed imaging strategy for screening BC.^37^ As a novel technology, digital breast tomosynthesis can address the limitations of mammography due to breast tissue overlap, thereby improving sensitivity and specificity.^38^ A recent review suggests that although mammography and magnetic resonance imaging are standard and cost-effective imaging methods for BC, in appropriate clinical settings, techniques such as digital breast tomosynthesis and contrast-enhanced mammography may be more cost-effective.^39^

 With the development of technology, AI and deep learning (DL) are gradually becoming important research directions in the field of cancer screening. The co-occurrence analysis of keywords in this study also shows that terms such as “machine learning” and “artificial intelligence” have frequently appeared in recent literature, indicating that they are becoming new research hotspots. The introduction of AI has brought significant potential for optimizing the process, improving the accuracy and efficiency of cancer screening, and representing the trend of cancer screening research towards interdisciplinary integration. AI, especially models including machine learning and DL, perform excellently in image recognition, lesion detection, and diagnostic assistance for various types of cancer. For example, in BC screening, AI technology has been used in triage procedures, imaging quality control, radiation dose optimization, and auxiliary diagnosis, significantly improving efficiency and accuracy.^40^ In LC screening, DL algorithms have shown high sensitivity in detecting lung nodules, and even in identifying smaller nodules.^41,42^ In colon cancer screening, AI-assisted colonoscopy detection models can improve the detection rate of polyps and adenomas.^43^ In terms of CC screening, AI models can interpret digital colposcopy images to improve the sensitivity and specificity of screening.^44^ In addition, AI can also assist doctors in diagnosing precancerous and malignant lesions based on biopsy sample testing, such as blood or tissue samples.^45,46^ Overall, AI and its embedding in the screening process may not only enhance the performance of existing screening technologies but also provide new automated and standardized tools for low-resource areas. Future related research can further explore the integration methods, algorithm performance optimization, cost-benefit analysis, and clinical promotion paths of AI in cancer screening strategies.

 Adherence has emerged as a key topic in the field of cancer screening, as evidenced by the burst of research keywords. Improving adherence is crucial for the effectiveness of cancer screening.^47^ However, adherence to cancer screening can be impacted by several barriers, such as inaccurate risk perception or lack of providers’ recommendations, as well as challenges related to transportation, language, and culture.^47^ Awareness of cancer and screening modalities is a key factor influencing participation in screening, as it influences beliefs, attitudes, and motivation. Simple and easily accessible testing procedures are often associated with higher adherence rates. Since cost remains a key barrier, publicly funded free screening initiatives coupled with mandatory insurance coverage or test fee exemptions would enhance screening availability.^48,49^ Enhancing adherence requires multidimensional collaboration among patients, providers, and healthcare organizations.

 Our study has certain limitations. Firstly, the ultimate results of the study are influenced by the selection of the Web of Science Core Collection database and the search queries used. Secondly, the quality of individual studies cannot be assessed through the bibliometric method. Additionally, because citations require time to accumulate, more recent articles generally show fewer citations compared to older ones.

 Nevertheless, this study systematically reviewed the scientific research achievements in the field of cancer screening, identifying research hotspots, key themes, and development trends. It can be seen from the clustering results that CRC, CC, LC, and BC are the cancers that received the highest attention. The screening methods mainly include fecal occult blood detection, HPV detection, and low-dose CT, which are highly consistent with the mainstream screening guidelines. It is worth emphasizing that this study not only provides cutting-edge trend references for academic research, but also has important practical guidance significance. Firstly, the research results can help clinical doctors, public health managers, and policy-makers identify which cancer screening methods have strong scientific support and practical feasibility, thereby optimizing the priority setting of screening projects. Secondly, by revealing the research focus and investment intensity of different countries and institutions, this study can provide a quantitative basis for policy-makers in resource allocation, regional cooperation, and technology selection. In addition, although emerging screening technologies such as AI and liquid biopsy have shown an increase in research enthusiasm, their clinical translation and inclusion guidelines are not yet sufficient. In the future, the validation and policy guidance of such technologies can be strengthened to promote effective transformation from scientific research achievements to practical applications.

 In summary, this study provides comprehensive and visual quantitative analysis results for the evolution, technological trends, and research resource distribution in the field of cancer screening. It not only has guiding value for future research but also provides strong data support for screening strategy optimization, evidence-based policy formulation, and public health intervention.

## Ethical issues

 Not applicable.

## Conflicts of interest

 Authors declare that they have no conflicts of interest.

## Data availability statement

 Data sharing is not applicable to this article as no datasets were generated or analysed during the current study.
